# Ultra-Wideband Positioning Sensor with Application to an Autonomous Ultraviolet-C Disinfection Vehicle

**DOI:** 10.3390/s21155223

**Published:** 2021-08-01

**Authors:** Shih-Ping Huang, Jin-Feng Neo, Yu-Yao Chen, Chien-Bang Chen, Ting-Wei Wu, Zheng-An Peng, Wei-Ting Tsai, Chong-Yi Liou, Wang-Huei Sheng, Shau-Gang Mao

**Affiliations:** 1 Graduate Institute of Commutation Engineering, National Taiwan University, Taipei 106, Taiwan; F06942082@ntu.edu.tw (S.-P.H.); d09942016@ntu.edu.tw (J.-F.N.); F05942018@ntu.edu.tw (Y.-Y.C.); F06942020@ntu.edu.tw (C.-B.C.); r07942009@ntu.edu.tw (T.-W.W.); D06942013@ntu.edu.tw (Z.-A.P.); weitingtsai@ntu.edu.tw (W.-T.T.); cyliou365tr@ntu.edu.tw (C.-Y.L.); 2 School of Medicine, National Taiwan University, Taipei 106, Taiwan; Wang-Huei_Sheng@gmail.com

**Keywords:** COVID-19, Ultraviolet-C, ultra-wideband, disinfection vehicle, wireless positioning

## Abstract

Due to the COVID-19 virus being highly transmittable, frequently cleaning and disinfecting facilities is common guidance in public places. However, the more often the environment is cleaned, the higher the risk of cleaning staff getting infected. Therefore, strong demand for sanitizing areas in automatic modes is undoubtedly expected. In this paper, an autonomous disinfection vehicle with an Ultraviolet-C (UVC) lamp is designed and implemented using an ultra-wideband (UWB) positioning sensor. The UVC dose for 90% inactivation of the reproductive ability of COVID-19 is 41.7 J/m^2^, which a 40 W UVC lamp can achieve within a 1.6 m distance for an exposure time of 30 s. With this UVC lamp, the disinfection vehicle can effectively sterilize in various scenarios. In addition, the high-accuracy UWB positioning system, with the time difference of arrival (TDOA) algorithm, is also studied for autonomous vehicle navigation in indoor environments. The number of UWB tags that use a synchronization protocol between UWB anchors can be unlimited. Moreover, this proposed Gradient Descent (GD), which uses Taylor method, is a high-efficient algorithm for finding the optimal position for real-time computation due to its low error and short calculating time. The generalized traversal path planning procedure, with the edge searching method, is presented to improve the efficiency of autonomous navigation. The average error of the practical navigation demonstrated in the meeting room is 0.10 m. The scalability of the designed system to different application scenarios is also discussed and experimentally demonstrated. Hence, the usefulness of the proposed UWB sensor applied to UVC disinfection vehicles to prevent COVID-19 infection is verified by employing it to sterilize indoor environments without human operation.

## 1. Introduction

### 1.1. System Description

To combat the highly infectious nature of the global health crisis, e.g., the COVID-19 pandemic disease, many intelligent-manufactured innovations have been launching to prevent infection by applying and integrating high-tech equipment. The wavelength range of 200–280 nm, which emits sufficient energy to shred the DNA or RNA of viruses, is effective in inhibiting bacteria, viruses, and fungi [1,2]. In addition, it can be used to sterilize in air and water or on the surface, showing the effectiveness of UVC when sterilizing indoors [3,4]. However, direct UVC exposure is harmful to the skin and eyes [5,6,7], and an immobile disinfection system cannot be used to sterilize in some areas hidden behind obstacles. Hence, the autonomous disinfection vehicle without human operation is suitable for this dirty, dull, and dangerous task [8].

In this study, an ultra-wideband (UWB) positioning sensor is applied to a disinfection vehicle with a UVC lamp [9] to enable the vehicle to navigate autonomously and thoroughly sterilize the GPS-denied environments without human operation.

### 1.2. Positioning Solutions

Conventional autonomous navigation is realized by restricting the paths to predetermined routes using guide strips [10], inertial measurement units [11], lasers [12], cameras [13], wireless fidelity (Wi-Fi) [14], Bluetooth [15], ultrasound [16], and UWB [17,18]. Guide strips are widely adopted in warehouses due to their low price and accuracy, but they are not flexible in high traffic areas and route-changing situations. Inertial measurement units are based on an accelerometer and gyroscope, and the error of this method accumulates over time. Cameras and light detection and ranging (LIDAR) using vision and lasers with simultaneous localization and mapping (SLAM) are popular solutions for autonomous navigation. Still, they are difficult to position in environments with little observation or repetitive features, such as large areas and long corridors [19,20]. Wi-Fi and Bluetooth use channel state information (CSI), received signal strength (RSS), or the signal to noise ratio (SNR) to fingerprint the characteristics of indoor environments [21,22]. The deploying density of base stations limits the positioning resolutions of these methods. The fingerprinting procedure must be repeated to maintain the accuracy of target localization when the environment changes. Ultrasound and UWB adopt time of arrival (TOA) or time difference of arrival (TDOA) to do multilateration and calculate the positions of tags [23,24]. The positioning accuracy depends on the time resolution of the propagating wave. Ultrasound is only suitable for line-of-sight (LOS) conditions, while UWB can be applied in non-line-of-sight (NLOS) conditions [25].

Multipath signals are generally caused by the radio signal being reflected by the ground, walls, or large metallic objects. The severe multipath in indoor environments can cause significant errors in RSS observations [26]. In contrast with RSS-based solutions, UWB can clearly distinguish LOS signals from multipath signals even in complex environments because of its precise time resolution [27]. The nanosecond-scaled Channel Impulse Response (CIR) provided by UWB chips can even be used to mitigate NLOS interference [25,28,29]. Moreover, recent developments in UWB technology have extended its interoperability and flexibility to provide cost-effective and robust solutions for autonomous navigation. Therefore, UWB technology is adopted in this work for wireless positioning and tracking.

The DWM1000 modules of Decawave are used as UWB devices in this work, and the operating frequency is 3.25–3.75 GHz with 500 MHz bandwidth [30]. The proposed UVC disinfection vehicle, using two UWB tags in the front and back, automatically generates and tracks the optimal path in a specific indoor environment. The location and direction of the vehicle can be found by positioning these two tags, and the direction toward the target can be calculated by using the positions of the vehicle and the target. By subtracting the two directions of the vehicle and the target, the turning angle of the vehicle can be obtained to move toward the target location. Accurate positions of the two tags are needed to make the vehicle move precisely toward the target or along the planned path. Hence, the UWB positioning system, with a TDOA algorithm, is applied to this UVC disinfection vehicle. Combining the UVC lamp and the UWB positioning system with the TDOA algorithm, the autonomous disinfection vehicle can thoroughly sterilize the specific indoor regions.

### 1.3. Traversal Path Planning

Traversal path planning procedures were also studied to autonomously navigate the entire indoor environment [31,32]. An intelligent mower using a UWB positioning system with path planning has been presented [33], and the primary method to traverse the map includes improved Boustrophedon cellular decomposition and the A* (called “A-star”) algorithm. However, this method is only optimized in simple conditions, and the repetition rate is high when there are numerous obstacles. Therefore, the generalized edge searching method is proposed to traverse all target nodes with the shortest paths in subareas connected by tunnels. For dead ends, all nodes are visited twice due to the limits of the vehicle size. The length of solution trace using a conventional method in [33] is longer than that using the proposed method, showing the advantage of the generalized edge searching method. By combining the UVC lamp and the UWB positioning system with the TDOA algorithm and the traversal path planning method, our autonomous disinfection vehicle can thoroughly sterilize the specific indoor regions.

## 2. Related Works

The use of a vision-based localization system for an autonomous mower was proposed in [34]. The camera toward the ground is used to analyze the features of each photo frame to determine the movement of the vehicle, and the angular acceleration sensor is added to eliminate the angle error caused by calculation. However, it can not be used on featureless surfaces such as environments with similar backgrounds. An indoor disinfection robot using LIDAR and the Gmapping algorithm to estimate the positions was introduced in [35]. The proposed hybrid path planning method uses the A* algorithm to find the global planning path and the Dynamic Window Approach (DWA) to update the path and avoid sudden obstacles. Although this previous work is flexible for various environments, it does not provide a traversal path planning method, which is vital for autonomous disinfection robots.

Using the Weighted Least-Square (WLS) method to find the approximated position as the input of the Taylor method was presented in [36]. It can achieve 12.6 cm accuracy with calibration but only 55.2 cm without calibration. This calibration process must be done in every new environment, which is labor-intensive and not practical for different application scenarios. Precise analysis of the TDOA wireless synchronization method was investigated in [37]. This synchronization method is helpful for the uploaded TDOA system, where tags blink in a fixed period to let anchors collect their messages and upload them to the cloud. However, the update rate is decreased as the number of tags increases. Therefore, it is not suitable to be applied for a large quantity of autonomous mobile robots (AMR). Comparisons between our proposed work and those previous studies are discussed and listed in Table 1.

This paper is organized as follows. Comparisons of this proposed work with other state-of-the-art published works are presented in Section 2. Section 3 discusses the UVC intensity of a cylindrical lamp with a D90 value, indicating the UVC dose for 90% inactivation of the reproductive ability of viruses. In Section 4, the UWB positioning system with anchor synchronization is introduced, and five different TDOA algorithms, including the Least-Square method, Chan method, Taylor method, GD method, and GD-Taylor method, are analyzed and compared. The edge searching method and the generalized traversal path planning procedure for autonomous navigation are presented in Section 5. Section 6 experimentally demonstrates the practical navigation of the UVC disinfection vehicle in a meeting room. The scalability of the proposed system to different scenarios is discussed in Section 7, and Section 8 draws conclusions and discusses future work.

## 3. Disinfection System Using UVC Lamp

UVC light is short-wavelength electromagnetic radiation that can destroy the reproductive ability of microorganisms or viruses by causing photochemical changes in nucleic acids. To inactivate the DNA or RNA of viruses, the UVC dose needs to be large enough. Furthermore, the impact of ultraviolet on different viruses has been investigated [38,39], showing that the average D90 value for several viruses is 47 J/m^2^. The UVC dose of 41.7 J/m^2^ is enough to inactivate 90% of reproductive ability, especially for the SARS-CoV-2 (COVID-19) virus.

**Figure 1:**
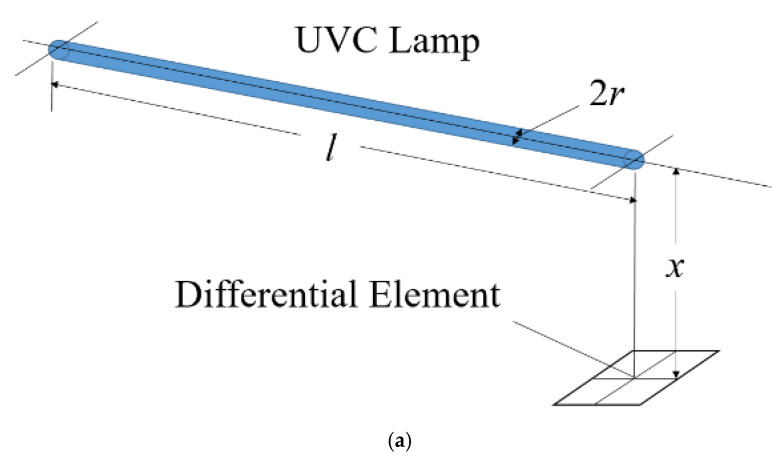

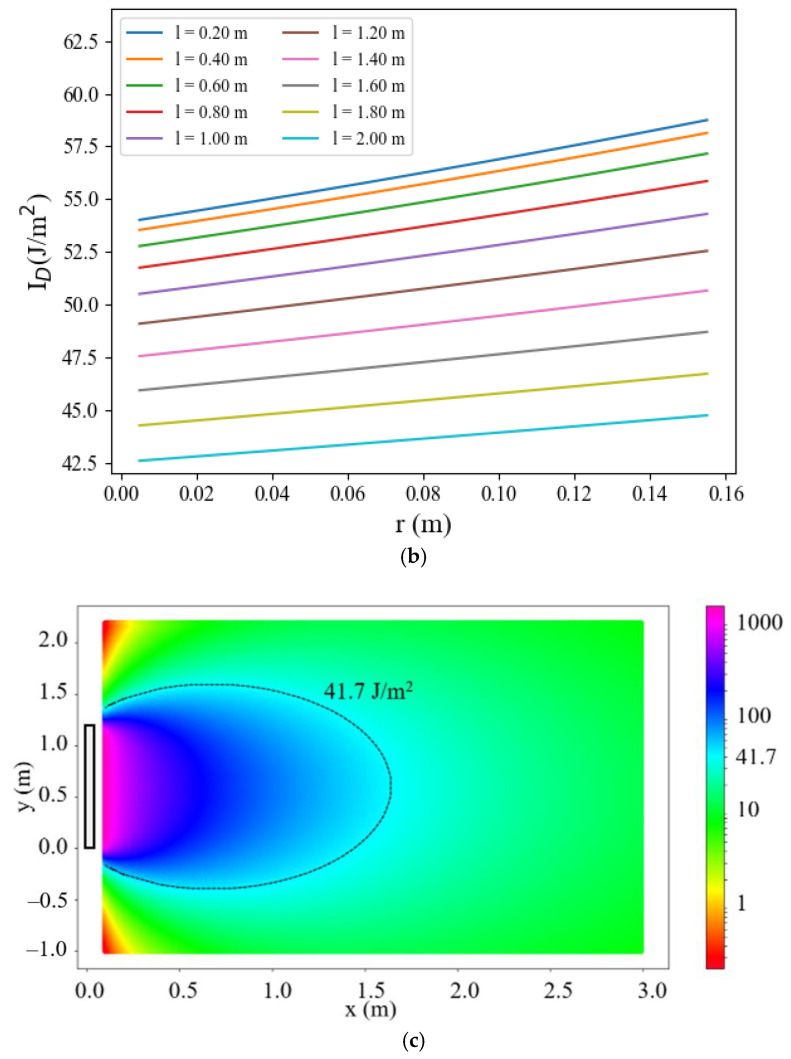
(**a**) Schematic diagram of the differential element and the cylindrical UVC lamp. The UVC intensity at (**b**) 1.5 m from the cylinder axis with various radii and lengths of the lamp, and (**c**) arbitrary points of the lamp with a 1.2 m length and 0.011 m radius. The exposure time is 30 s, and the total UVC power is 40 W. The color bar shows the value of light intensity in the unit J/m^2^, and the dashed line represents 41.7 J/m^2^.

Hence, UVC intensity field analysis is necessary to accurately determine the dose to be delivered to the microorganisms or viruses. In Figure 1a, the intensity at a point outside a UVC lamp can be computed using the radiation factor from a finite cylinder to a differential planar element, when the normal axis of the element is perpendicular to the cylinder axis and located axially at one end of the cylinder. The intensity caused by a segment of a UVC lamp with a length of *l* can be expressed, according to [40], as (1) and (2):

(1) $$I_{D}(l)=EF_{t}(l)t/2\pi rl,$$

(2) $$F_{t}(l)=\frac{L}{\pi D}[\frac{1}{L}\tan^{-1}(\frac{L}{\sqrt{D^{2}-1}})+\frac{X-2D}{\sqrt{XY}}\tan^{-1}(\sqrt{\frac{X(D-1)}{Y(D+1)}})-\tan^{-1}(\sqrt{\frac{D-1}{D+1}})]$$

where

$$D=\frac{x}{r},\;L=\frac{l}{r},\;X={\left(1+D\right)}^{2}+L^{2},\;Y={\left(1-D\right)}^{2}+L^{2},$$

*x* is the perpendicular distance from the lamp to the point, *l* is the length of the lamp segment, *r* is the radius of the lamp, *E* is the total UVC power radiated from the lamp, *t* is the total exposure time, F_t_(*l*) is the radiation factor, and I_D_(*l*) is the intensity caused by UVC at the point in Figure 1a.

**Figure 2:**
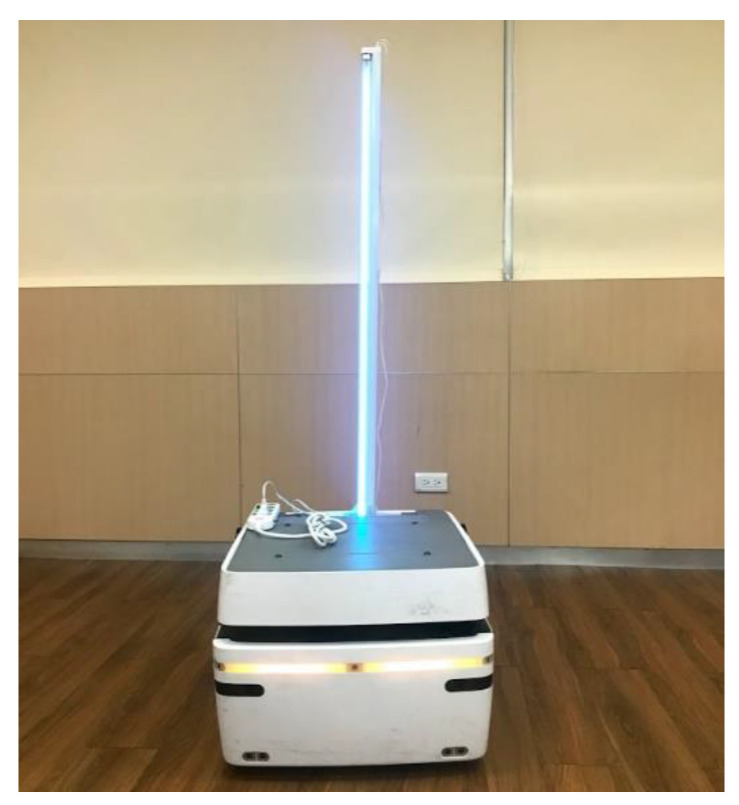
Photo of the disinfection vehicle with UVC lamp.

By using (1) and (2), the intensity of UVC light from a cylindrical lamp with a different *r* and *l* and at arbitrary points with a different *x* and *y* can be drawn as Figure 1b,c, respectively. In Figure 1b, the UVC light focuses on the main beam, which is the perpendicular direction from the center of the UVC lamp, with a smaller length and larger radius of the lamp. In Figure 1c, the lamp is installed at *x* = 0, *y* = 0−1.2 m, the radius *r* is 0.011 m, the exposure time *t* is 30 s, and the UVC power from the lamp *E* is 40 W. Results show that the 41.7 J/m^2^ UVC intensity (D90 value of COVID-19) can be achieved within the dashed-line region (Figure 1c), which covers x = 0−1.6 m for the main beam direction. Hence, the sterilization procedure can be completed in 30 s within a 1.6 m radius using the disinfection vehicle with a UVC lamp, as shown in Figure 2.

## 4. UWB Positioning System with TDOA Algorithm

### 4.1. Modified TWR and Anchor Synchronization

The UWB positioning system, using two-way ranging (TWR) and NLOS mitigation, was proposed in our previous work [25]. However, the positioning interval increases drastically when there are a large number of devices that need to be located. Therefore, the TDOA algorithm, with a constant positioning interval, is applied in this work.

**Figure 3:**
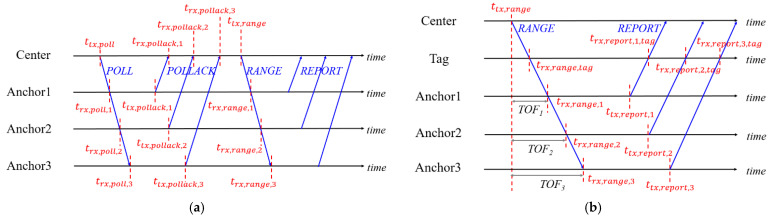
(**a**) The modified TWR between the Center and anchors. (**b**) TDOA with anchor synchronization.

The synchronization of anchors is necessary to implement the UWB positioning system with the TDOA algorithm because the clock frequency ratio (CFR) and transmitting time offset vary in different devices. The CFR is defined as the ratio of timestamps calculated by two devices in the same interval. For ${\mathrm{Anchor}}_{i}$ in Figure 3a, the CFR can be written as:

(3) $$r_{Anchor,i}=\frac{t_{rx,range,i}-t_{rx,poll,i}}{t_{tx,range}-t_{tx,poll}}$$

The time of flight (TOF) between the Center, which is the initiator of communication, and ${\mathrm{Anchor}}_{i}$ based on the clock of the Center can be written as:

(4) $$T_{i}=\frac{(t_{rx,pollack,i}-t_{tx,poll})-\frac{t_{tx,pollack,i}-t_{rx,poll,i}}{r_{Anchor,i}}}{2}$$

This is the modified TWR, which is introduced to calculate the distance based on the clock of the Center. In Figure 3b, the Tag receives all signals from the Center and Anchors. By receiving the poll and range from the Center, the CFR of a Tag can be written as:

(5) $$r_{tag}=\frac{t_{rx,range,tag}-t_{rx,poll,tag}}{t_{tx,range}-t_{tx,poll}}$$

Then, the synchronized timestamp of ${\mathrm{Anchor}}_{i}$ is:

(6) $$t_{rx,report,i,tag}^{\prime }=t_{rx,report,i,tag}-r_{tag}\times T_{i}-(t_{tx,report,i}-t_{rx,range,i})\frac{r_{tag}}{r_{Anchor,i}}$$

These synchronized timestamps can be used in TDOA calculation with the received timestamp from the Center. In this procedure, the tags only receive signals. Therefore, the number of tags can be unlimited.

### 4.2. TDOA Positioning Algorithms

**Figure 4:**
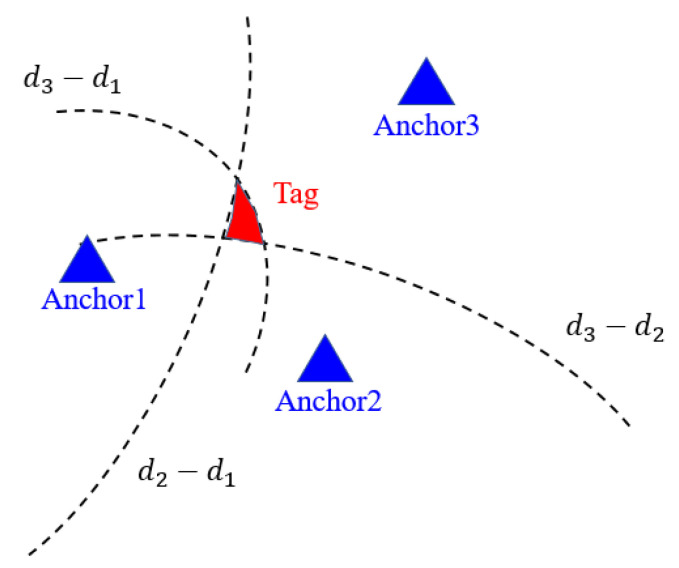
The hyperbolas may intersect in a region instead of a point. The position of a Tag needs to be estimated.

With the synchronized timestamp differences of the Center and Anchors, the differences of the distances can be obtained by multiplying the time resolution and light speed in the air. However, the hyperbolas generated by the differences of distances may intersect in a region instead of a point, as shown in Figure 4. The position of a Tag needs to be estimated by using the TDOA positioning algorithm.

Assume that the coordinates of the $i_{th}$ anchor are [$x_{i}$, $y_{i}$], and the coordinates of the estimated position of the Tag are [*x*, *y*]. The measured difference of distances is $d_{ij}$ between the $i_{th}$ anchor and the $j_{th}$ anchor, and the distance between the estimated Tag and the $i_{th}$ anchor is $d_{i}$. Then, the purpose of the algorithm is to find the minimum of the loss function as below:

(7) $$f=\sum_{i>j}(\sqrt{{\left(x-x_{i}\right)}^{2}+{\left(y-y_{i}\right)}^{2}}-\sqrt{{\left(x-x_{j}\right)}^{2}+{\left(y-y_{j}\right)}^{2}}-d_{ij}{)}^{2}$$

One of the traditional methods is the Least-Square (LS) closed-form solution. The relationships of $d_{ij}$,$\;d_{i}$, and [$x_{i}$, $y_{i}$] can be expressed in matrix form [41]:

(8) Aθ=b

where

(9) $$A=\left[\begin{array}{ccc} x_{2}-x_{1} & y_{2}-y_{1} & d_{21}\\ x_{3}-x_{1} & y_{3}-y_{1} & d_{31}\\ & \vdots & \\ x_{n}-x_{1} & y_{n}-y_{1} & d_{n1} \end{array}\right],\;\theta =\left[\begin{array}{c} x\\ y\\ d_{1} \end{array}\right],\;b=\left[\begin{array}{c} x_{2}^{2}+y_{2}^{2}-x_{1}^{2}-y_{1}^{2}-d_{21}\\ x_{3}^{2}+y_{3}^{2}-x_{1}^{2}-y_{1}^{2}-d_{31}\\ \vdots \\ x_{n}^{2}+y_{n}^{2}-x_{1}^{2}-y_{1}^{2}-d_{n1} \end{array}\right]$$

The solution can be written as:

(10) $$\theta ={(A^{T}A)}^{-1}A^{T}b$$

Another solution is the Chan method. The method is based on a twice LS solution, and it is widely used in TDOA estimation [42,43]. However, the estimated position is not precise enough by only using LS and the Chan method.

The Taylor method is a recursive method with an initial position. The displacement in each iteration can be calculated by [43,44]:

(11) $$\delta_{Taylor}=\left[\begin{array}{c} \Delta x\\ \Delta y \end{array}\right]={(G^{T}Q^{-1}G)}^{-1}G^{T}Q^{-1}h$$

where

$$G=\left[\begin{array}{cc} (x_{1}-x)/d_{1}-(x_{2}-x)/d_{2} & (y_{1}-y)/d_{1}-(y_{2}-y)/d_{2}\\ (x_{1}-x)/d_{1}-(x_{3}-x)/d_{3} & (y_{1}-y)/d_{1}-(y_{3}-y)/d_{3}\\ \vdots & \;\vdots \\ (x_{1}-x)/d_{1}-(x_{n}-x)/d_{n} & (y_{1}-y)/d_{1}-(y_{n}-y)/d_{n} \end{array}\right],$$

$$h=\left[\begin{array}{c} d_{21}-(d_{2}-d_{1})\\ d_{31}-(d_{3}-d_{1})\\ \vdots \\ d_{n1}-(d_{n}-d_{1}) \end{array}\right],$$

$$Q=\left[\begin{array}{cccc} \mathrm{std}(d_{21}) & 0 & \cdots & 0\\ 0 & \mathrm{std}(d_{31}) & & 0\\ \vdots & & \ddots & 0\\ 0 & 0 & 0 & \mathrm{std}(d_{n1}) \end{array}\right].$$

By recursively changing the estimated position of a Tag until the displacement is small enough, the accuracy of the Tag position can be improved. However, at some locations, the estimated position of a Tag using the Taylor method is far from the real position due to the small determinant of $G^{T}Q^{-1}G$.

The Gradient Descent (GD) method is also a recursive method with an initial position [45,46]. The displacement comes from the partial differentials of (7), which are calculated as:

(12) $$\begin{array}{c} f_{x}=2\sum_{i>j}\left[(\sqrt{{\left(x-x_{i}\right)}^{2}+{\left(y-y_{i}\right)}^{2}}-\sqrt{{\left(x-x_{j}\right)}^{2}+{\left(y-y_{j}\right)}^{2}}-d_{ij}\right)\\ \times (\frac{x-x_{i}}{\sqrt{{\left(x-x_{i}\right)}^{2}+{\left(y-y_{i}\right)}^{2}}}-\frac{x-x_{j}}{\sqrt{{\left(x-x_{j}\right)}^{2}+{\left(y-y_{j}\right)}^{2}}})] \end{array}$$

(13) $$\begin{array}{c} f_{y}=2\sum_{i>j}\left[(\sqrt{{\left(x-x_{i}\right)}^{2}+{\left(y-y_{i}\right)}^{2}}-\sqrt{{\left(x-x_{j}\right)}^{2}+{\left(y-y_{j}\right)}^{2}}-d_{ij}\right)\\ \times (\frac{y-y_{i}}{\sqrt{{\left(x-x_{i}\right)}^{2}+{\left(y-y_{i}\right)}^{2}}}-\frac{y-y_{j}}{\sqrt{{\left(x-x_{j}\right)}^{2}+{\left(y-y_{j}\right)}^{2}}})] \end{array}$$

(14) $$\delta_{GD}=\left[\begin{array}{c} \Delta x\\ \Delta y \end{array}\right]=\left[\begin{array}{c} -f_{x}\\ -f_{y} \end{array}\right]$$

Instead of adding the displacement directly to [*x*, *y*], using an adaptive gradient is helpful for finding the minimum of the loss function [47]. The GD method gives a better accuracy with the cost of more computing time than the Taylor method. The GD-Taylor method is proposed by combining these methods. Considering both the gradient and Taylor series, the displacement can be modified as:

(15) $$\delta_{GD-Taylor}=\delta_{GD}+\delta_{Taylor}$$

The detail of this method is shown in Algorithm 1, and the distance differences are calculated in Step 1. Then, Step 2–3 initializes the weight of the adaptive gradient and the position of the Tag. The main loop (Step 4–15) iteratively moves the estimated tag position. Step 5 determines the distances from anchor positions to the estimated tag position in this loop, and Step 6 calculates **δ***_Taylor_* and **δ***_GD_* by (11) and (14). Step 7 and Step 8 determine **δ***_GD-Taylor_* and *weight*, respectively. Step 9 and Step 10 apply the modified adaptive gradient method, and Step 11 upgrades the iteration number. Step 12–14 check the norm of displacement for the early stopping of the main loop. Finally, the estimated tag position is output in Step 16.
**Algorithm 1** Function GD-Taylor()**Input**Locations of anchors (*x*_1_, *y*_1_), (*x*_2_, *y*_2_), …, (*x_n_, y_n_*)
Received timestamps *t*_1_, *t*_2_, …, *t_n_*
Maximal iteration time *max_iter*
Initial location (*x_init_, y_init_*)**Output**Estimated location of tag (*x_t_, y_t_*)1Calculate *d*_21_, *d*_31_, …, *d*_*n*1_ by multiplying light speed and 
time resolution to (*t*_2_ − *t*_1_), (*t*_3_ − *t*_1_), …, (*t_n_* − *t*_1_); 2Set *weight* to 10^−10^; 3Set (*x*, *y*) to (*x*_*init*_, *y*_*init*_);4**while***times* < *max_iter*
**do**5*d*_1_, *d*_2_, …, *d_n_* are the distances from anchors to (*x*, *y*);6use (8) and (11) to calculate **δ***_Taylor_* and **δ***_GD_*;7Set **δ***_GD-Taylor_* to (**δ***_Taylor_* + **δ**_*GD*_);8Set *weight* to (*weight* + **δ**_*GD-Taylor, x*_^2^ + **δ**_*GD-Taylor, y*_^2^);9Set *x* to (*x* + **δ**_*GD-Taylor, x*_ /(*weight*)^1/2^);10Set *y* to (*y* + **δ**_*GD-Taylor, y*_ /(*weight*)^1/2^);11*times*++;12**if** ((**δ**_*GD-Taylor, x*_^2^ + **δ**_*GD-Taylor, y*_^2^)/*weight*)^1/2^ < 0.001 **then**13**break**14 **endif**15**endwhile**16**return** (*x*, *y*)

The displacement of the GD-Taylor method is large at first for fast convergence, which is contributed by the Taylor method. After several iterations, the estimated position is close to the real position, and the displacement is small enough to satisfy Step 12 for early stopping. In addition, Step 8–10 control the displacement to avoid data explosion caused by the Taylor method. Thus, the GD-Taylor method possesses the advantages of both the Taylor and GD methods in calculating speed and accuracy.

### 4.3. Simulation and Measurement

**Figure 5:**
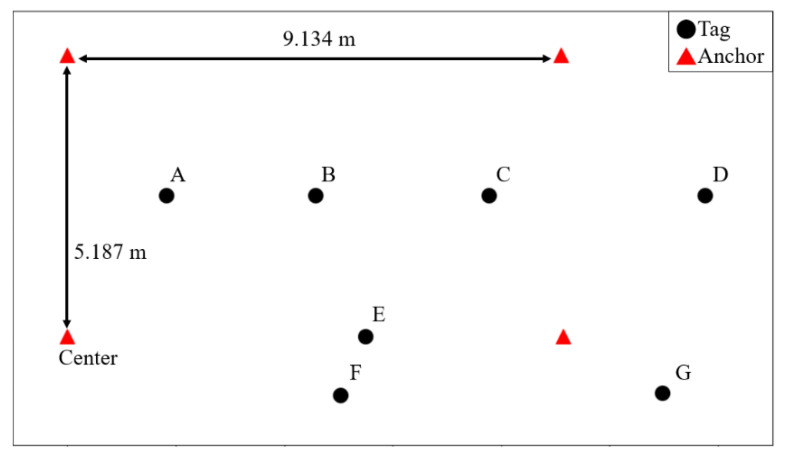
The deployment of anchors and tags in simulation and measurement of the UWB positioning system with the TDOA algorithm. The black circles represent the real positions of tags, and the red triangles represent the positions of Anchors, including the Center.

The UWB positioning system, with four anchors for simulation and measurement, is shown in Figure 5. The positions of A, B, and C are inside the rectangle of anchors, while the positions of D, E, F, and G are not. In the simulation, the actual distance between each Tag and Anchor is calculated by real positions in Figure 5. The simulated distance is the actual distance added by a delta distance. The delta distance is generated by a normal distribution with a mean value of 0 and a standard deviation of 0.1 m. After calculating the differences of these simulated distances, the estimated positions of tags can be calculated using different algorithms. Root mean square errors (RMSEs) are calculated in 500 simulations for each tag position, and the simulated result of different positioning algorithms is shown in Table 2. The Taylor method gives the wrong estimation at some tag positions, such as C and D, but the GD-Taylor method still possesses accurate results. The simulated RMSE using the GD-Taylor method is below 20 cm for A, B, C, and E and is below 30 cm on average.

A measurement using the UWB TDOA positioning system with the deployment in Figure 5 is carried out, and the measured result is shown in Table 3. The measured results are similar to the simulated results, and the GD-Taylor method still gives the best accuracy on average. The accuracy of A, B, C, D, and E using the GD-Taylor method is below 30 cm, implying that tags can be identified even if they are placed within 30 cm of each other. In addition, the RMSEs of tag positions within the rectangle region of anchors are smaller than those outside the rectangle region of anchors. Therefore, the anchors should be installed in appropriate positions to cover all tag positions when using the TDOA positioning system, and the Taylor method should be modified to the GD-Taylor method to increase the accuracy of estimated positions. The average calculating time for each position using different positioning methods is shown in Table 4. The result shows that the estimation using the GD-Taylor method is four times faster than that using the GD method, which is due to the combination of the GD and Taylor methods. Therefore, the GD-Taylor method is useful in practical TDOA positioning systems for both accuracy and calculating speed.

## 5. Traversal Path Planning Using Generalized Edge Searching Method

Before planning a path, a map should be expressed as nodes with a respective x, y, and function. The forbidden regions and obstacles are composed of inaccessible nodes. The regions that need to be traversed are composed of target nodes, while the other regions are composed of accessible nodes.

Traversal path planning aims to visit all target nodes in a map with the shortest path. The Hamilton path is the path that goes through every target node just once. Finding a Hamilton path with the smallest weight is an NP-hard problem, and the path may not exist. In addition, the time complexity of the exhaustive method is O(n!), which is too big for practical usage.

A feasible solution is the backtracking method. First, set an initial node and search for neighbors. Second, recursively visit a neighbor until all neighbors are visited or inaccessible. Third, cancel the visit, go back to the last node, and visit another neighbor. By recursively doing these steps, a Hamilton path can be found, and the time complexity is $O(x^{n})$, where x is the number of neighbors for one node, and n is the total number of target nodes. However, for the map with more than 100 target nodes, the consuming time of path planning is still too long.

### 5.1. Edge Searching Method

The edge searching method is proposed to optimize the consuming time of path planning. To avoid splitting too many regions in a map, traversing along the edge is considered when using the backtracking method. The way to traverse the whole region in a few decisions is to visit along the corner and edge by selecting the neighbor with the minimal number of available neighbors. In this work, the eight nearest nodes with straight and oblique links are called neighbors, and the four nearest nodes with only straight links are called straight neighbors. The variable *node.available* is the number of unvisited and reachable neighbors for the node, and the details of the edge searching method are shown in Algorithm 2.
**Algorithm 2** Function EdgeSearching(*visiting_node*)**Input**The current trace *visited_trace*
The current node *visiting_node*
Total number of target nodes *total_length*
The length of solution *solution_length***Output**The trace of solution *solution_trace*1**if***solution_length* is *total_length*
**then**2**return**3**endif**4**if** (length of *visited_trace*) is *total_length*
**then**5Set solution_length to (length of *visited_trace*);6**return***visited_trace*7**endif**8Set *min_value* to 10;9**for** *node* **in** neighbors of *visiting_node* **do**10**if***node* not visited and *node.available* < *min_value* **then**11Set *min_value* to *node.available*;12**endif**13**endfor**14**for** *node* **in** neighbors of *visiting_node* **do**15**if***node* not visited and *node.available* is *min_value* **then**16Visit *node* and add *node* to *visited_trace*;17**for** *neighbor_node* **in** neighbors of *node* **do**18neighbor_node.available--;19**endfor**20EdgeSearching(*node*);21**for** *neighbor_node* **in** neighbors of *node* **do**22neighbor_node.available++;23**endfor**24Unvisit *node* and delete *node* **in** *visited_trace*;25**end if**26**end for**

In Algorithm 2, the termination is called in Step 1–3, and Step 4–7 set the termination condition and return the solution when all target nodes are visited. Step 8 initializes the *min_value*, and Step 9–13 set the *min_value* to the minimal available among neighbors of the visiting node. The main loop (Step 14–26) visits the neighbors of the visiting node with minimal *available* and calls the function itself to traverse the map. Step 15 chooses the unvisited neighbors of the visiting node with minimal *available*, and Step 16 visits the node. The *availables* of neighbors near the node decrease in Step 17–19 because the node is visited. Step 20 calls the function EdgeSearching (node) to further visit the next node. The *available* of neighbors near the node increase in Step 21–23, and the node is unvisited in Step 24 because the current trace meets a dead end in Step 20.

**Figure 6:**
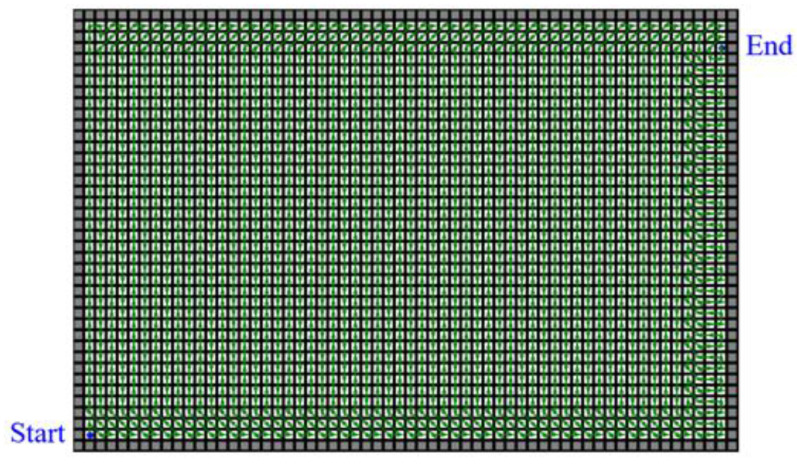
The solution using the edge searching method in a 60 × 40 m^2^ region. The gray grids represent inaccessible nodes, and the white grids represent the target nodes. The optimal trace is drawn as green arrows, and the blue points show the beginning and the end of the trace.

The edge searching method chooses the neighbor along the edge and reduces the time complexity of path planning to $O(x^{\sqrt{n}})$. Moreover, it can usually find a Hamilton path in a few iterations, so it is helpful to figure out an optimal solution even if the number of target nodes is more than 1000. The solution using the edge searching method in a simple region is shown in Figure 6.

However, the edge searching method may fail when there is no Hamilton path in the region. Therefore, some target nodes must be dealt with first or visited twice, such as tunnels and dead ends. A tunnel is composed of nodes that contain only two straight neighbors, and a dead-end is a node with only one straight neighbor plus a tunnel or not. A Hamilton path may not exist when the target nodes contain dead ends or tunnels. Therefore, all target nodes should be classified as subareas, tunnels, and dead ends, ensuring an optimal solution.

### 5.2. Generalized Traversal Path Planning

**Figure 7:**
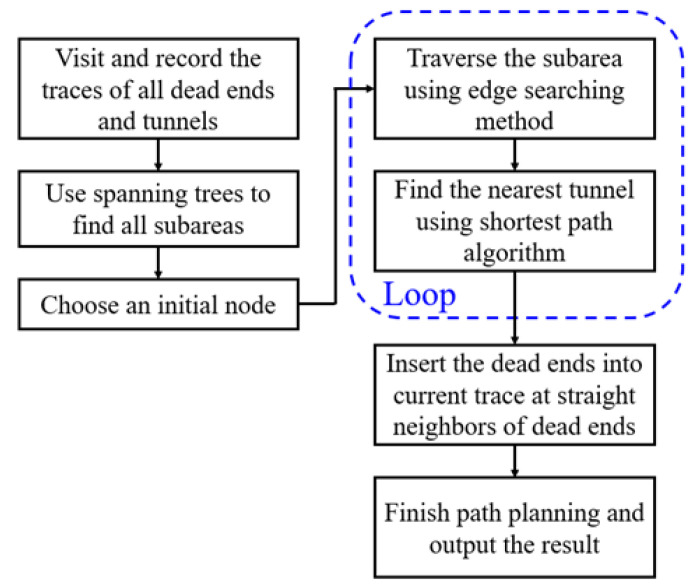
The generalized traversal path planning procedure using the edge searching method and shortest path algorithm.

The generalized traversal path planning procedure, using the edge searching method, is shown in Figure 7. Through this procedure, Hamilton paths in all subareas can be found and connected by tunnels or shortest paths. The tunnels are found and traversed in the loop, and the dead ends are added after other nodes are visited.

**Figure 8:**
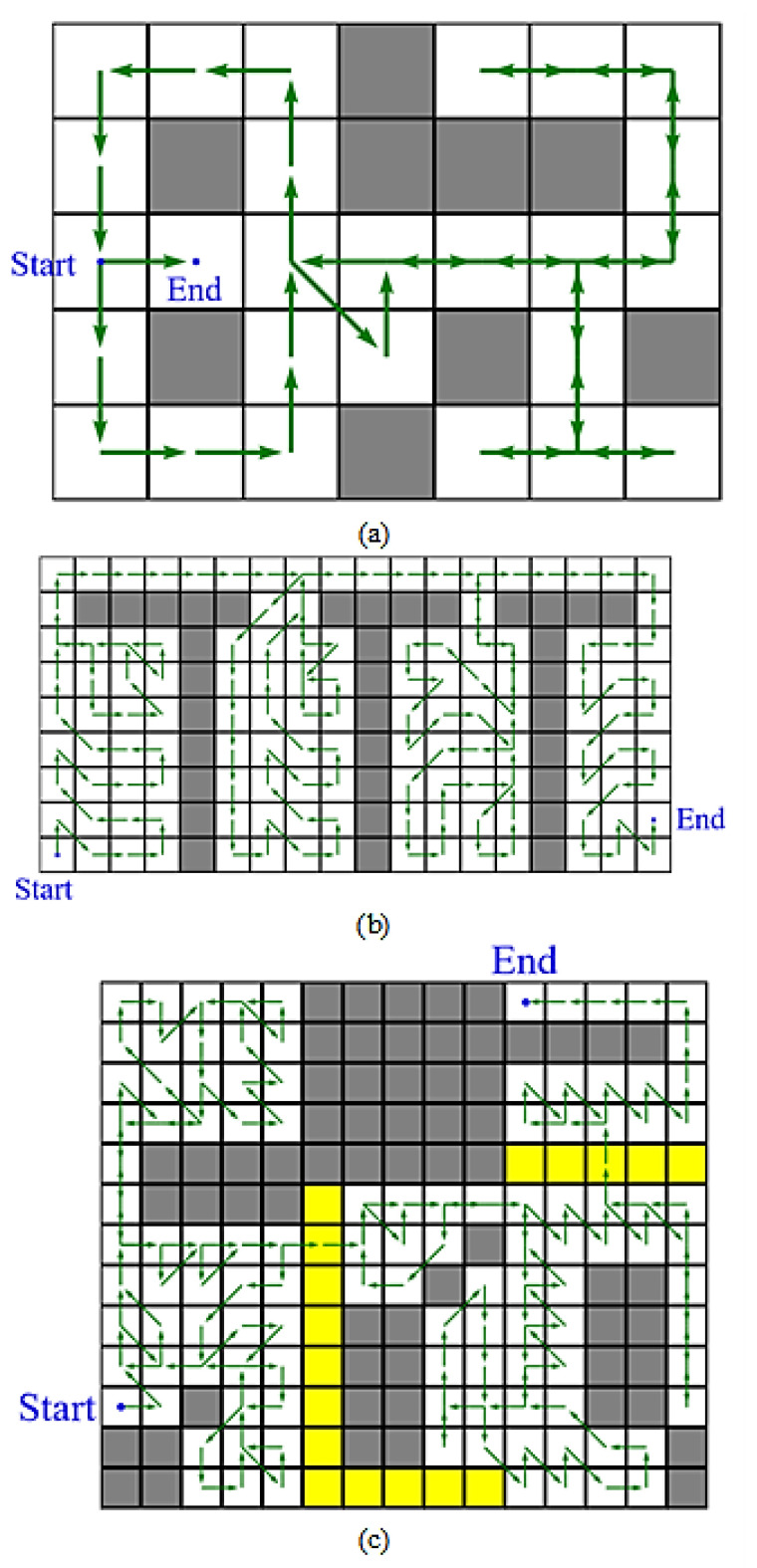
The solutions using generalized traversal path planning for different situations. The target nodes, inaccessible nodes, and accessible nodes are drawn as white grids, gray grids, and yellow grids, respectively. The traces of solutions are drawn as green arrows, and the blue points show the beginnings and the ends of the traces. (**a**) A simple example with tunnels and dead ends. (**b**) An example for four rooms and one corridor. (**c**) A complicated example with two yellow regions composed of accessible nodes.

Some solutions of examples are shown in Figure 8. In Figure 8a, the dead ends are visited twice when necessary, and the solution is the optimal trace in this situation. In Figure 8b, there are four rooms and one corridor. The corridor is considered as a tunnel, and the four rooms are classified as subareas. The subareas are traversed only once and then connected to the nearest tunnels with the shortest paths, and the tunnels are all visited once for optimization in this situation. In Figure 8c, two yellow regions are composed of accessible nodes, which are not necessary to be visited. The solution trace connects the subareas through these two regions by the shortest paths, and the accessible nodes are visited for only two nodes in this solution. These results show that the generalized traversal path planning, using the edge searching method, helps find optimal solutions when the map is expressed as target nodes, inaccessible nodes, and accessible nodes.

## 6. Demonstration

**Figure 9:**
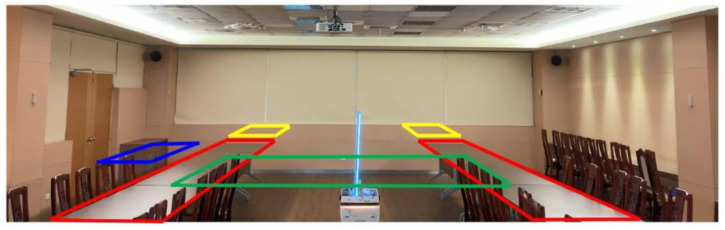
The disinfection vehicle and the environment of the experiment.

**Figure 10:**
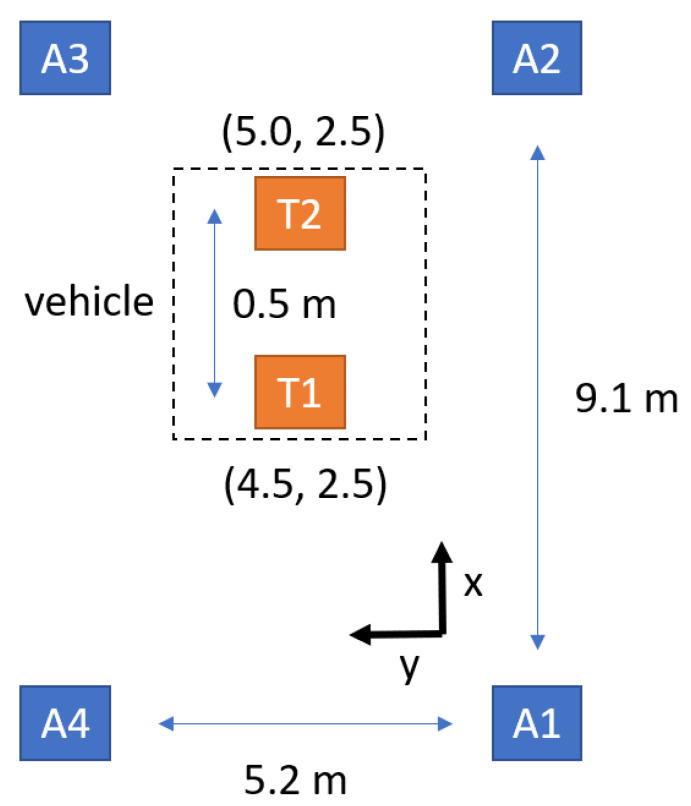
Setups for checking the estimated direction accuracy.

**Figure 11:**
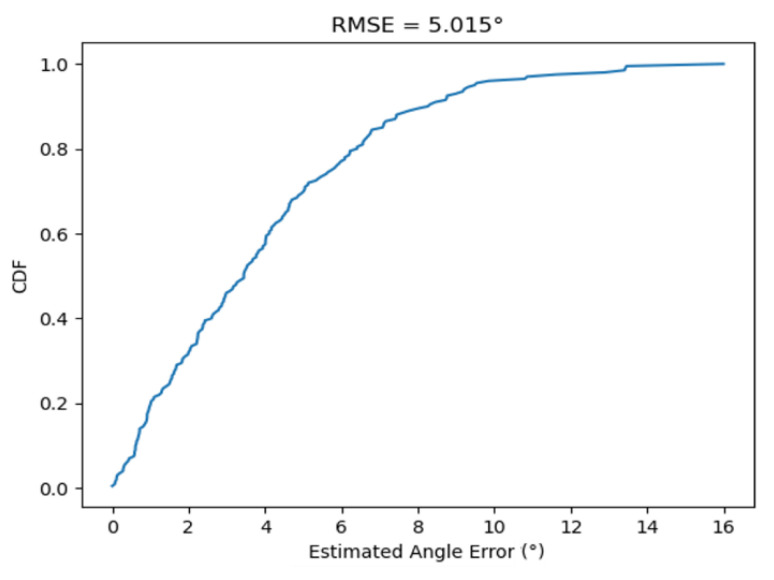
CDF of the direction error in the experiment.

Combining UVC sterilization and the UWB positioning sensor, the autonomous disinfection vehicle (Figure 2) is implemented. In Figure 9, an experiment is carried out using the disinfection vehicle with two UWB tags installed at the front and back area and four UWB anchors in a 9.1 × 5.2 m^2^ meeting room. The distance between the two tags is 0.5 m, which is needed to compute the vehicle’s direction, as shown in Figure 10. First, the vehicle with two tags is placed at the center of the room to check the estimated direction accuracy. After recording 200 estimated directions, the root-men-square-error (RMSE) of estimated directions is found to be 5.015°, and the cumulative density function (CDF) of estimated directions is depicted in Figure 11.

**Figure 12:**
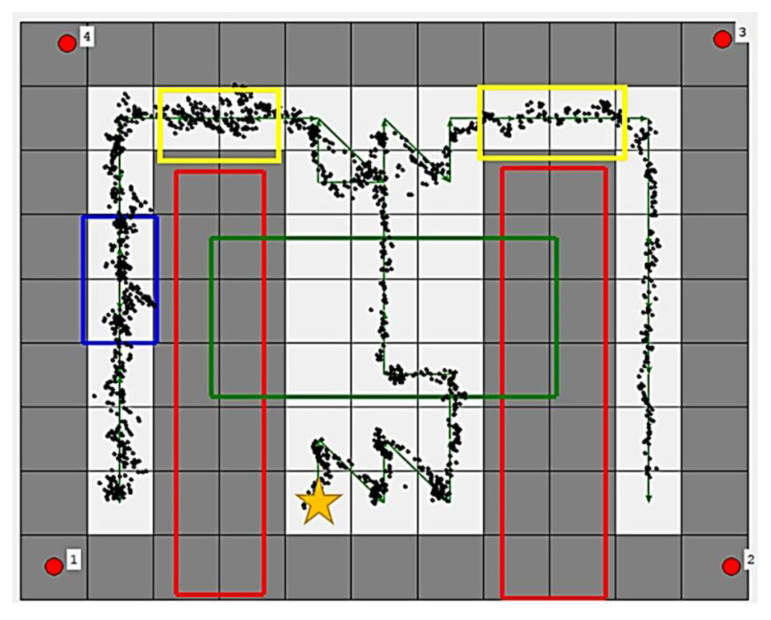
The planned path and positioning results in the experiment. The green arrows represent the path and its direction.

The map is divided into 0.8 × 0.8 m^2^ grid nodes, as shown in Figure 12. The red rectangles contain the inaccessible nodes representing the obstacles in the environment, which are tables in this meeting room. The green rectangle is the accessible region that does not need to be disinfected but allows the vehicle to pass. The blue rectangle is the working region where the vehicle stays for 30 s to provide a 41.7 J/m^2^ UVC dose for 90% inactivation of the reproductive ability of the COVID-19 virus. The yellow rectangles are the slow down regions where the vehicle moves slowly for safety.

**Figure 13:**
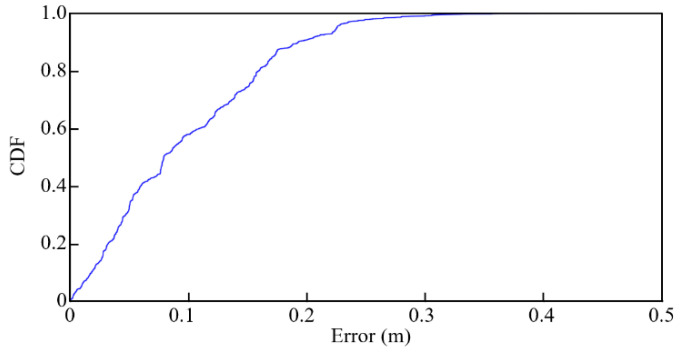
CDF of error in the experiment.

The path in the green line is generated using the traversal path planning algorithm to match the regional restrictions. The vehicle starts at the yellow star and stops at the bottom of the right aisle. The black points show the average estimated positions of the two UWB tags. The distances between the black points and the ideal trace are calculated to verify the accuracy of the positioning system. The average error is only 0.1 m, and the RMSE is 0.13 m. The CDF of error is shown in Figure 13. It is evident that over 50% of points are below 0.1 m error, and about 90% points are below 0.2 m error. The results demonstrate the usefulness of the disinfection vehicle with the proposed algorithms and positioning systems.

## 7. Discussion

### 7.1. Choice of Grid Size

**Figure 14:**
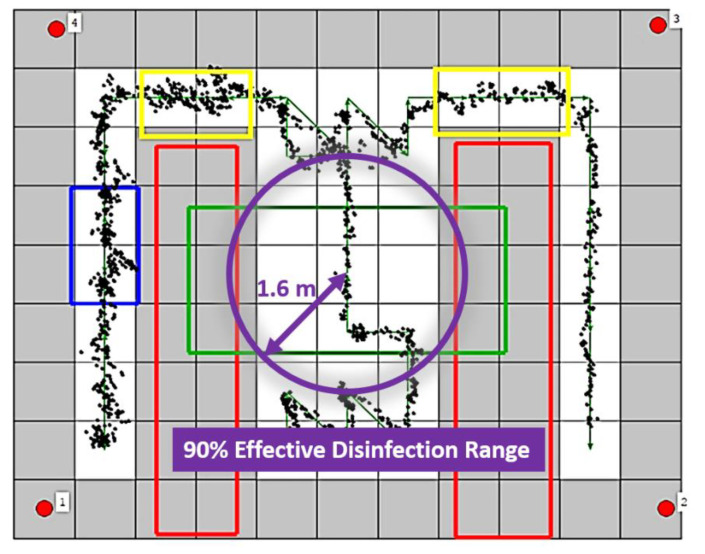
Disinfection coverage at a point.

In the actual scenario, the grid size should be considered carefully. It should be larger than the disinfection vehicle to avoid collisions but small enough to be able to adapt to a complex environment. Further, the grid size should not exceed the disinfection range. If the grid size has been appropriately chosen, the disinfection vehicle can go through a room along the most efficient path, proposed in Section 5.2, and complete the sterilization process in a single planned path. In this paper, a UVC lamp with a 1.6 m disinfection range is used. Thus, the grid size is evaluated to be 0.8 × 0.8 m^2^, which is smaller than the 1.6 m disinfection range of the UVC lamp, as illustrated in Figure 14. Thus, a 100% disinfection percentage in this room can be achieved.

### 7.2. Different Scenarios

**Figure 15:**
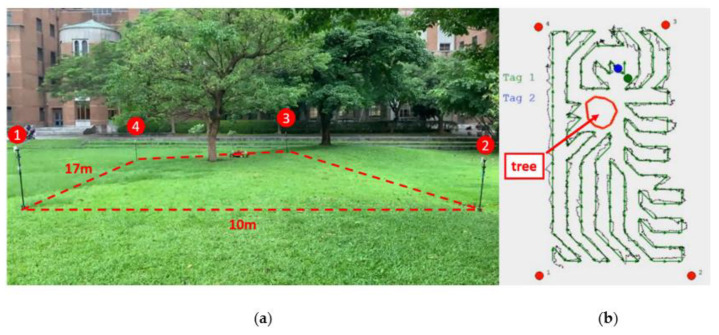
Autonomous mower scenario. (**a**) The lawn mowing area of 10 × 17 m^2^ with a tree inside. (**b**) Planned path and the actual trace of the auto mower.

Another application scenario of the proposed autonomous vehicle system is also presented in this paper. Figure 15a shows a desired lawn mowing area of 10 × 17 m^2^ with a tree inside. Four UWB anchors are placed at the corner of the region, and two UWB tags are deployed at the front and back areas of the mower. The area of the tree has been configured as a forbidden region. The grid size is chosen to be 1.0 × 1.0 m^2^. Figure 15b shows the planned path in the green line and the actual trace of the mower in the black line. This result demonstrates that the proposed autonomous vehicle system with the UWB positioning system and traversal path planning algorithm can be applied to various application scenarios.

### 7.3. Autonomous Vehicles for Different Surfaces

The adaptability of an autonomous vehicle for various surfaces depends significantly on the motion system to keep the robot moving efficiently in different environments. The wheel-type mechanism can run fast on a flat surface but cannot cross obstacles smoothly. The track-type mechanism has high adaptation for crossing obstacles, but it consumes much power, and the speed is relatively slow. The wheel-track hybrid robot with four modes can climb up to a 25° slope [48]. The wheel-legged robot that can transform from a wheel-shape into a leg-shape was proposed in [49]. This robot can walk upstairs or pass through deep gaps in the leg-shape mode. In Figure 15a, this proposed UWB positioning system is successfully applied to the autonomous lawnmower with high positioning accuracy even on rugged terrain. Hence, the usefulness of our solution is verified for various autonomous vehicles.

## 8. Conclusions

In this study, an autonomous disinfection vehicle with a UVC lamp is designed and implemented by using a UWB positioning sensor with the TDOA algorithm. The UVC light intensity is analyzed, and the results show that the D90 value can be achieved for a 1.6 m distance at 30 s exposure time. The anchor synchronization method for UWB positioning is introduced, and the GD-Taylor method for the TDOA algorithm is proposed. The simulated and measured results show that the GD-Taylor method possesses high accuracy and short computing time. A traversal route in the shortest path is established using the generalized traversal path planning procedure with the edge searching method. By deploying the autonomous disinfection vehicle in a 9.1 × 5.2 m^2^ room, the average positioning error is only 0.1 m. Experimental results validate the effectiveness of the proposed algorithm and the performance of the autonomous disinfection vehicle. Thus, the UVC disinfection vehicle demonstrates the effectiveness of autonomous vehicles and is suitable for sterilization without human assistance in indoor environments. Furthermore, novel artificial intelligence algorithms for swarm AMRs applications and autonomous unmanned aerial vehicles with three-dimensional wireless positioning techniques are currently under investigation and will be presented in the future.

## Figures and Tables

**Table 1:** Features comparisons for UWB-related works.

Paper	Average Accuracy (cm)	Supported AMR Quantity	Update Rate (Hz)
This work	28.7	infinite	50
[37]	36.0	1170	5
[36]	55.2 (no calibration) 12.6 (with calibration)	1000	32

**Table 2:** RMSEs of Tag positions using different algorithms in simulation.

Tag	LS	Chan	Taylor	GD	GD-Taylor
A	111.963	9.699	0.114	0.118	0.118
B	0.164	0.748	0.136	0.149	0.147
C	127.223	8.051	3.723E + 17	0.131	0.130
D	45.700	5.614	4.914E + 08	0.506	0.452
E	185.042	0.187	0.162	0.173	0.171
F	66.120	3.658	0.211	0.220	0.218
G	0.924	2.182	0.797	0.808	0.773
Average	76.691	4.305	5.318E + 16	0.300	0.287

Unit: m.

**Table 3:** RMSEs of Tag positions using different algorithms in measurement.

Tag	LS	Chan	Taylor	GD	GD-Taylor
A	15.952	8.881	0.183	0.167	0.168
B	2.245	0.701	0.132	0.123	0.124
C	10.648	3.602	6.654E + 07	0.134	0.131
D	15.990	4.036	4.560E + 17	0.483	0.277
E	2.414	0.148	0.168	0.159	0.161
F	133.374	4.844	0.343	0.338	0.339
G	0.805	1.969	0.611	0.594	0.601
Average	25.867	3.454	6.645E + 16	0.279	0.257

Unit: m.

**Table 4:** Average calculation time using different algorithms.

	LS	Chan	Taylor	GD	GD-Taylor
Average Calculating Time	0.0868	0.6141	0.4129	12.4678	3.1685

Unit: ms.
